# Supplementary material for: Tracking the contribution of inductive bias to individualised internal models
Source: PLoS Comput Biol. 2022 Jun 22;18(6):e1010182. doi: 10.1371/journal.pcbi.1010182 (PMC9255757; doi:10.1371/journal.pcbi.1010182)
Supplement: S1 Appendix — (PDF) [file pcbi.1010182.s001.pdf]

S1 Appendix – A brief introduction to infinite Hidden Markov Models

Hidden Markov Models are used to model discrete-time sequences of observations by introducing a latent state which is not directly observable (hidden) and link them with probabilistic transitions. Suppose we are modelling the behaviour of our neighbour. We identify some of the chores they are doing during the day, such as hoovering, sleeping and working. These activities would be the states of our dynamical system and the noises we hear in our house are the observations from which we can guess what the neighbour is doing. Some noises are clearly indicative of the activity (e.g. hoovering) but the absence of noise can be accounted for by multiple different states (e.g. sleeping and working). However, these states would be different because hoovering is less likely to occur during sleeping and more likely during the day or after working. If we would like to accurately predict our neighbour's behaviour and be able to plan for the loud noises, we might need more states to be introduced, such as cooking, eating, showering. One general problem with hidden Markov models is that if we are uncertain a priori of the number of states governing the dynamics, we may introduce unnecessary states or be unable to differentiate states when we should.

The infinite hidden Markov model introduces the concept of having infinitely many latent states. An important observation is, however, that in finite time there can only be finitely many states occurring. The Hierarchical Dirichlet Process-Hidden Markov Model [1] makes learning infinitely many different states possible by linking the states in a hierarchical manner. The model states that both transition probabilities into other states and observation distributions come from a common prior. For these reasons, we can group the unobserved states as ”one” during the execution of our sampling or other inference algorithm. When the neighbour starts to hold book clubs, we introduce a new state, called book club and estimate its parameters: what noises we hear and what could happen before and after.

The key strength of such non-parametric Bayesian models (assuming infinitely many groups) is that we will only add new groups (in our case, states) when there is sufficient

evidence to do so. In practice this means that the number of hidden states we believe occurred during a period of time can only grow if there is more evidence as time passes. Having listened to the neighbour for one week, the infinite hidden Markov model will assume just a handful of states but having spent years next door we may even learn about when holiday season is for the neighbour's piano teacher.

References

[1] Teh YW, Jordan MI, Beal M, Blei D. Hierarchical dirichlet processes. Journal of the American Statistical Association. 2006; p. 1–41. doi:10.1017/CBO9781107415324.004.
