# Supplementary material for: Tracking the contribution of inductive bias to individualised internal models
Source: PLoS Comput Biol. 2022 Jun 22;18(6):e1010182. doi: 10.1371/journal.pcbi.1010182 (PMC9255757; doi:10.1371/journal.pcbi.1010182)
Supplement: S2 Appendix — (PDF) [file pcbi.1010182.s002.pdf]

S2 Appendix – Optimal prediction and the LATER model

A rational objective for human participants in a prediction task could be to minimize the expected response time. Let us denote the probabilities of the possible outcomes of a prediction task as p_i, the corresponding subjective probabilities (predictions) with $\hat{p_i}$. Then, according to the LATER model, we have:

$$\mathbb{E}_{p,r}(RT) = \sum_i p_i \cdot \mathbb{E}_r\left(\frac{\tau_0 - log(\hat{p_i})}{r}\right) = c_1 + c_2 \cdot \sum_i p_i \cdot (-\log(\hat{p_i}))$$

where we group quantities not depending on $\hat{p_i}$ into constants c_1, c_2.

Note that the we quantity we obtained at the end is the cross-entropy between the actual probabilities and the subjective probabilities.

We can take this further, note that the entropy of the actual probabilities, $H(p_i)$ does not depend on the participant's predictions, therefore we can also subtract this:

$$\sum_i p_i \cdot (-\log(\hat{p_i})) + p_i \cdot \log(p_i) = D_{KL}(P, \hat{P})$$

This is the Kullback-Leibler divergence between the actual probabilities (P) and the subjective probabilities ($\hat{P}$). Therefore expected response times are minimized exactly when the subjective probabilities match the actual probabilities.
