# Supplementary figures and images for: Tracking the contribution of inductive bias to individualised internal models
Source: PLoS Comput Biol. 2022 Jun 22;18(6):e1010182. doi: 10.1371/journal.pcbi.1010182 (PMC9255757; doi:10.1371/journal.pcbi.1010182)

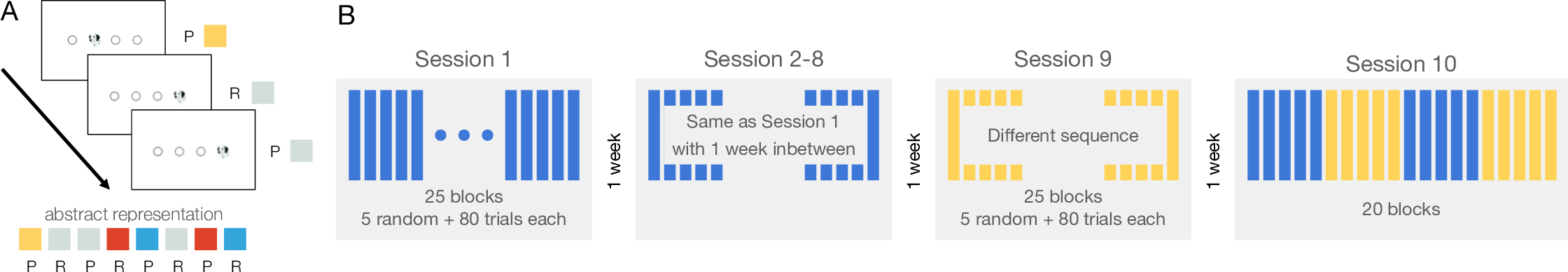

Supplement: S1 Fig — A Experimental stimuli and abstract representation used in the paper. B Design of the experiment. The experiment consisted of ten sessions, separated by a one-week delay. On Days 1–8, participants performed the ASRT task with sequence 1 throughout 25 blocks (5 epochs) each sessions. On Day 9, an interfering sequence (sequence 2) was introduced. Both sequences were tested on Day 10 with blocks of 5 alternating. (TIF) [file pcbi.1010182.s003.tif]

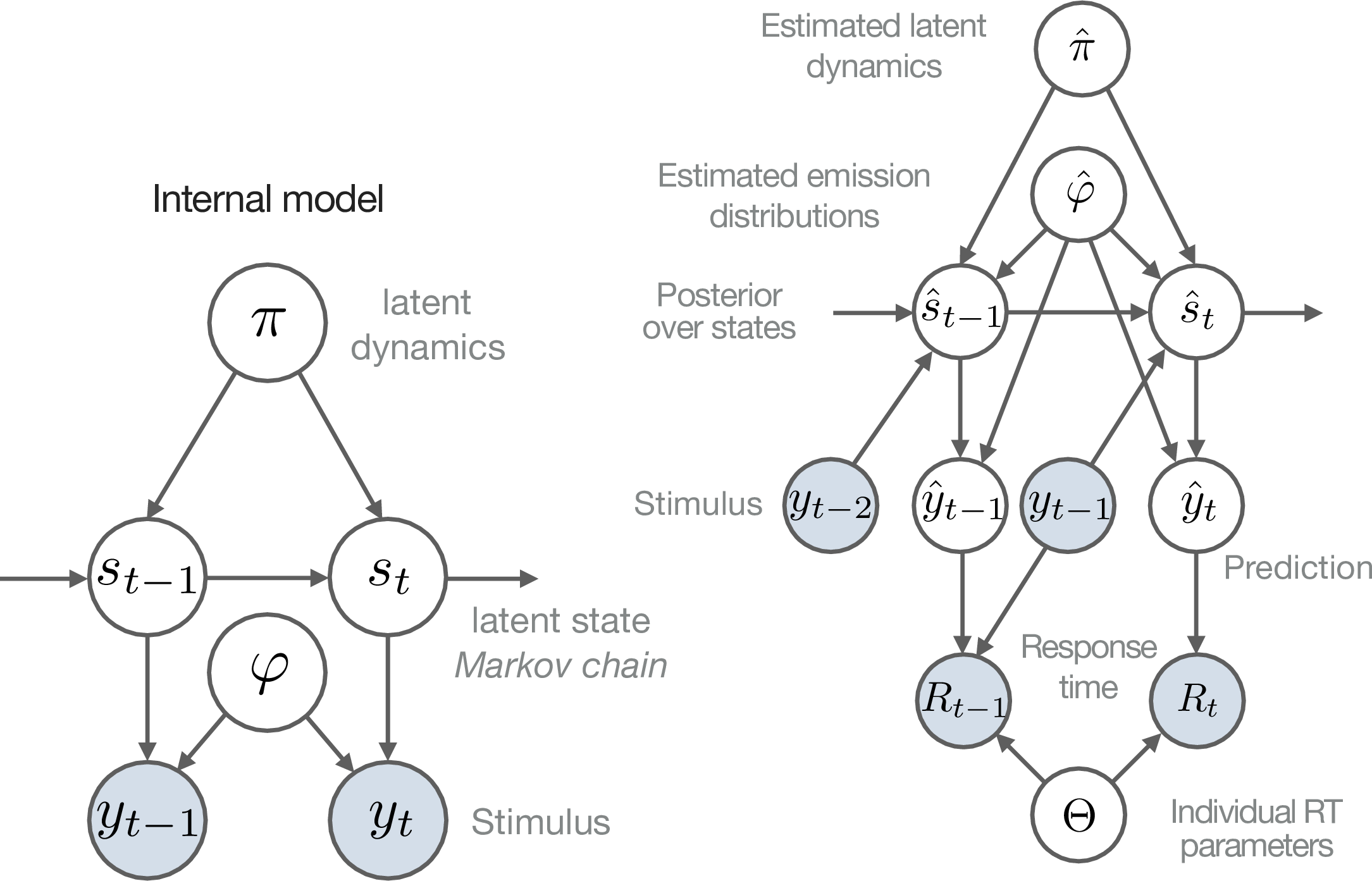

Supplement: S2 Fig — Left: Internal model, generative model of the sequence assumed by the participant. Right: generative model of behaviour. (TIF) [file pcbi.1010182.s004.tif]

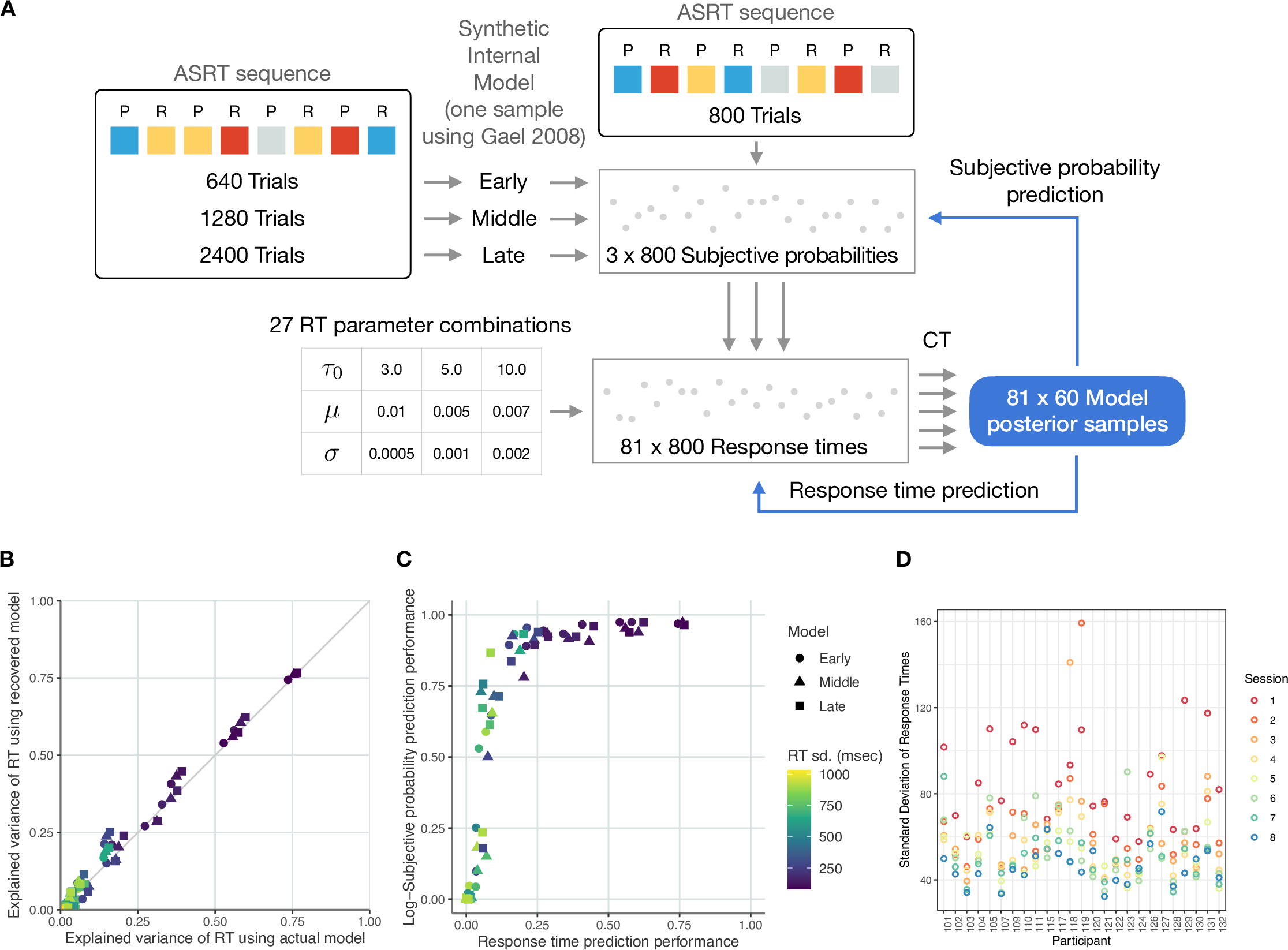

Supplement: S3 Fig — A We first sampled three versions of synthetic internal models using the original iHMM inference method in [33]. The internal models of the synthetic participants differ in their experience (as how many ASRT trials they had seen)—resulting in an “early”, “middle” and “late” model. Then, we generated subjective probability values for each model on a new set of ASRT stimuli (holding the pattern sequence intact). B Results of our synthetic data experiment. Performance is measured as the amount of variance in response times (R2). We ran our inference method for 81 synthetic datasets with different parameter settings (symbols with different colors and shapes). We use the same number of response times as with the human participants to recover the internal models. Symbol colours correspond to the response time standard deviation. The result shows that while the response time prediction may be at a lower level, the latent predictive probabilities can still be inferred with relatively high accuracy. This shows the inference method can recover the latent structure from a generated response time sequence. C Predictive performance (R2) of the actual internal model of the synthetic participant vs the predictive performance of the inferred internal model of the same synthetic participant. The inferred model is evaluated on train data sets (same as on panel B). D Standard deviations of response times of individuals in the first eight experimental sessions. (TIF) [file pcbi.1010182.s005.tif]

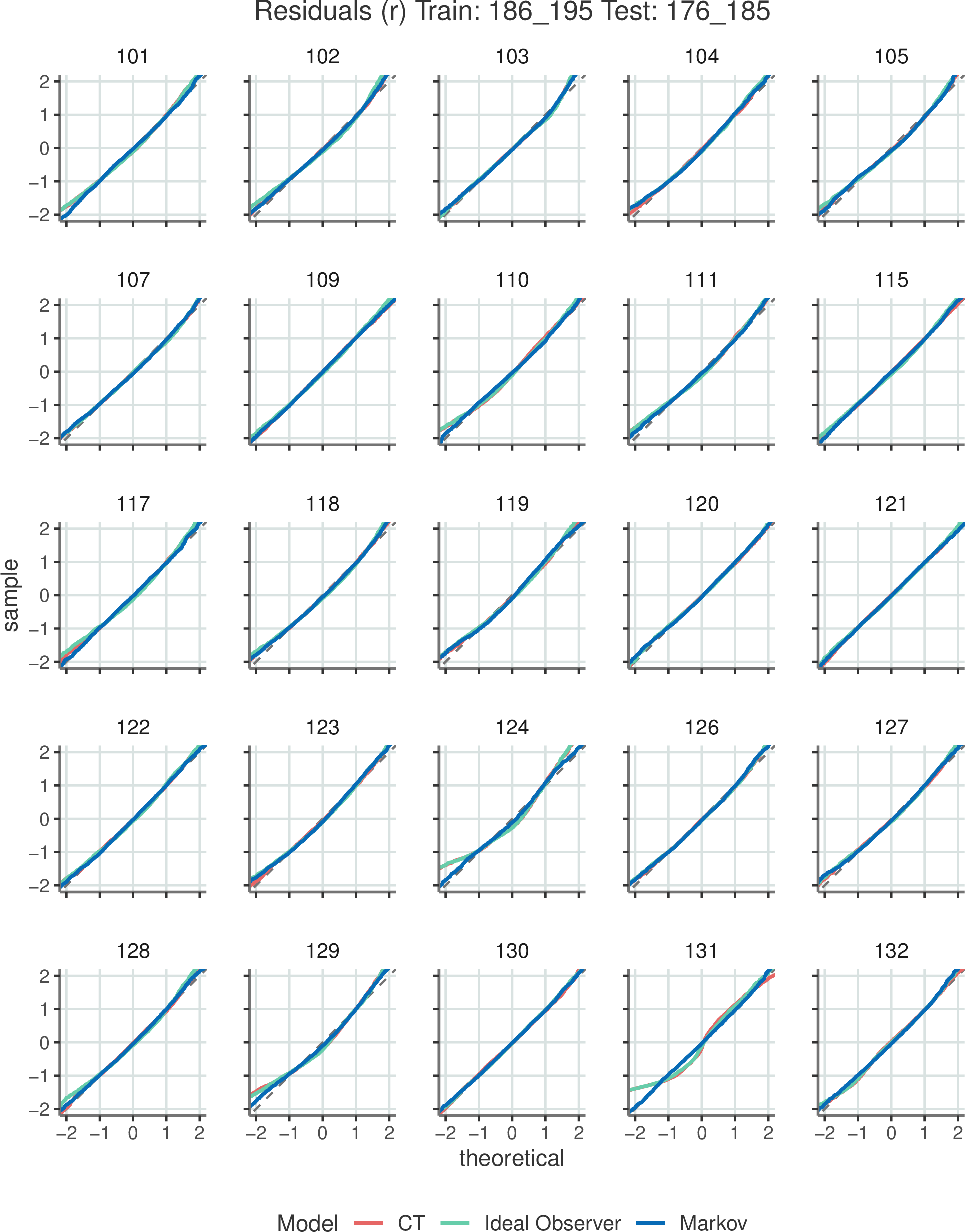

Supplement: S4 Fig — Quantiles are computed from the response times and predicted subjective probabilities with quantiles of the expected normal distribution for the analysed models (red, CT; green, ideal observer; blue, Markov), also known as QQ-plots. Participant-by-participant shows that the empirical distribution of the r parameter on a test set is approximately normal with a few exceptions (see participants 124 131 CT and Ideal Observer models), thus validating model assumptions. (TIF) [file pcbi.1010182.s006.tif]

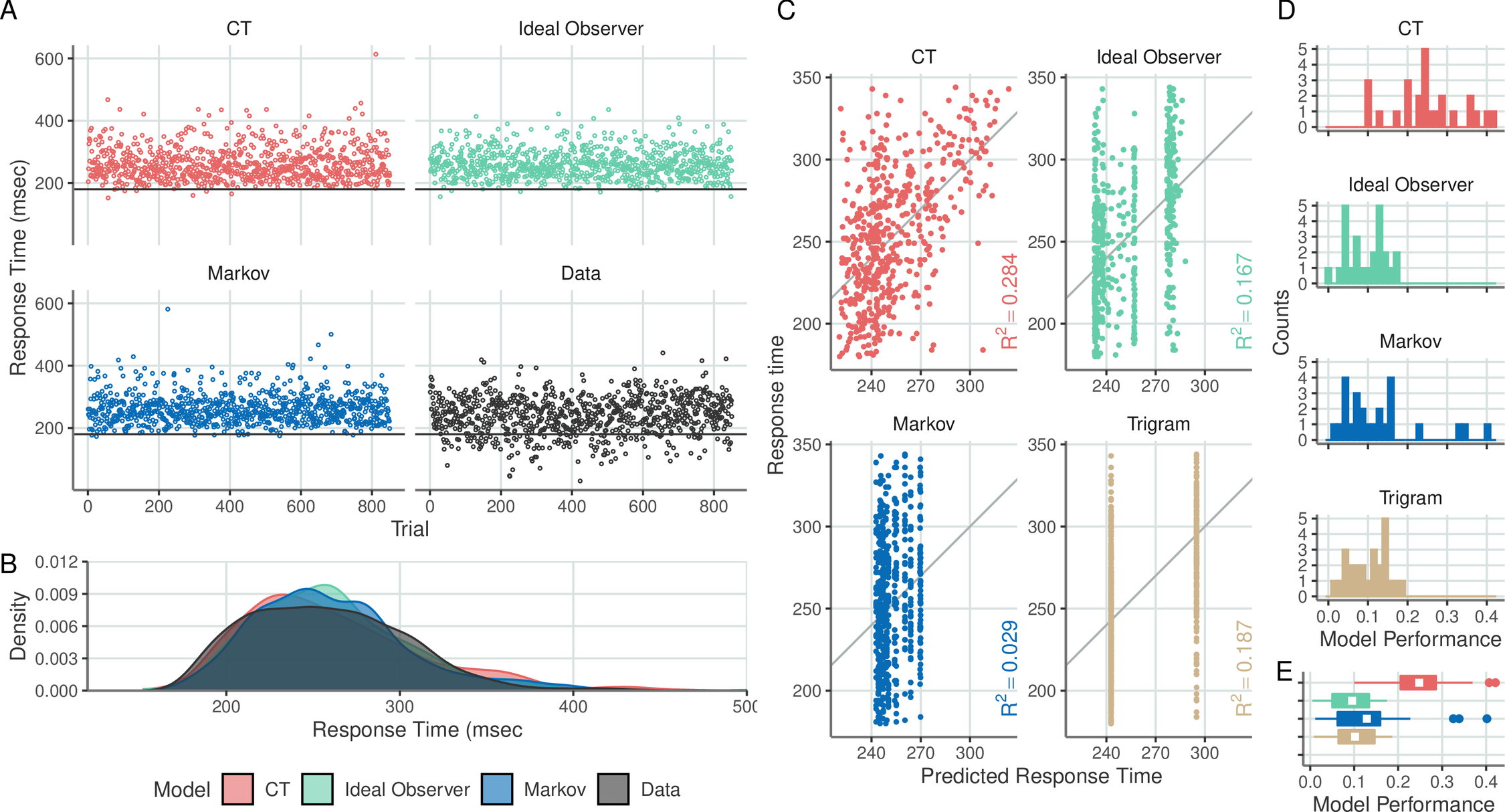

Supplement: S5 Fig — A Response time samples generated from different models and the original Data for participant 119. B The density plots of the point clouds in A. C Predicted response times (mean of maximum a posteriori estimates for each model averaged over the model samples) vs actual response times. Response times outside mean ±3 s.d. are omitted for visual clarity. In contrast with panel A, the x coordinates are best predictions rather than random samples, hence their spread is much smaller. D Histogram of model predictive performances on Day 8. 9 Box plots of model performance distributions, data same as panel D. (TIF) [file pcbi.1010182.s007.tif]

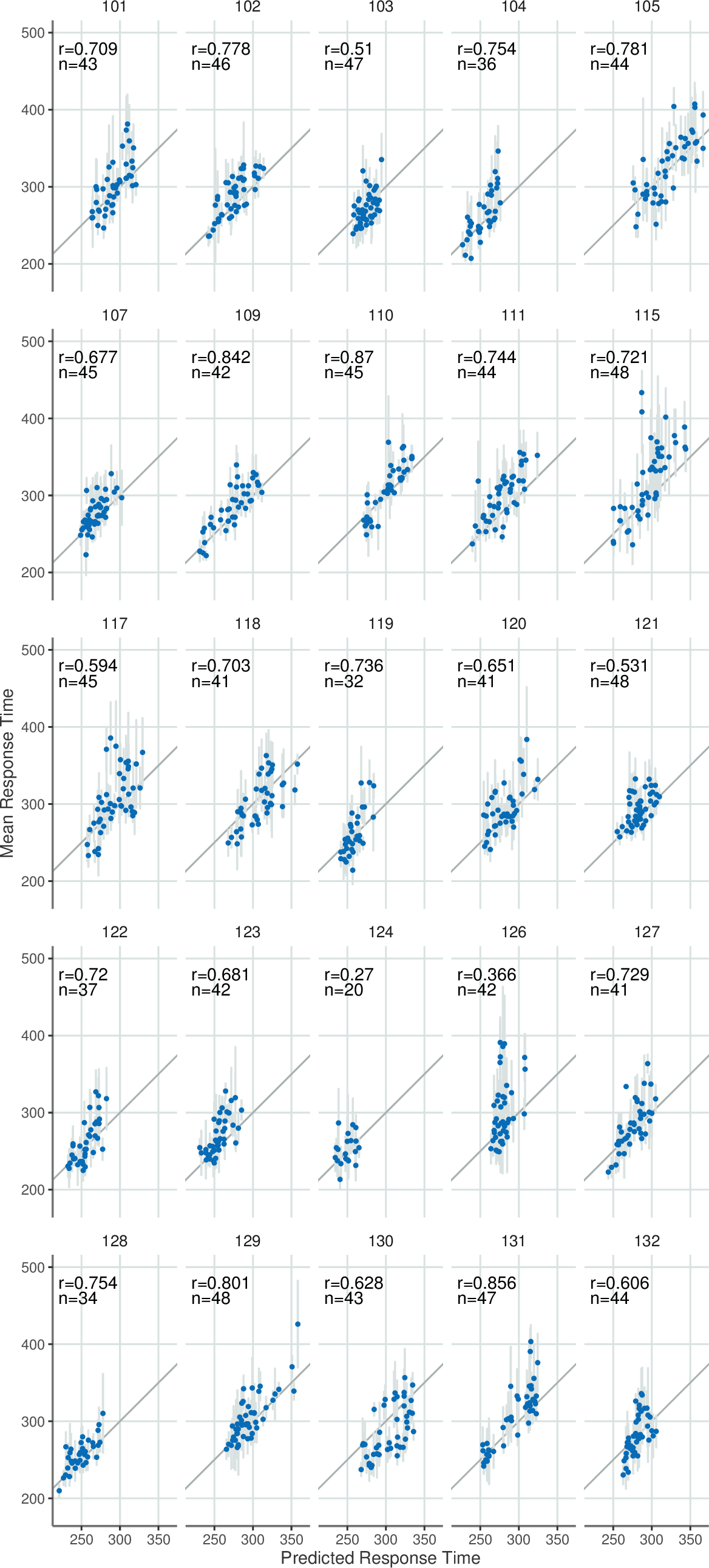

Supplement: S6 Fig — Predictions are on the test set on Day 8 of the experiment grouped by three element sequences for each participant separately (each dot corresponds to one possible three-element sequence). Only those sequences were included which had at least 5 measured correct response times in order to limit the standard error over the measured response time mean. Error bars show 2 s.e.m. (TIF) [file pcbi.1010182.s008.tif]

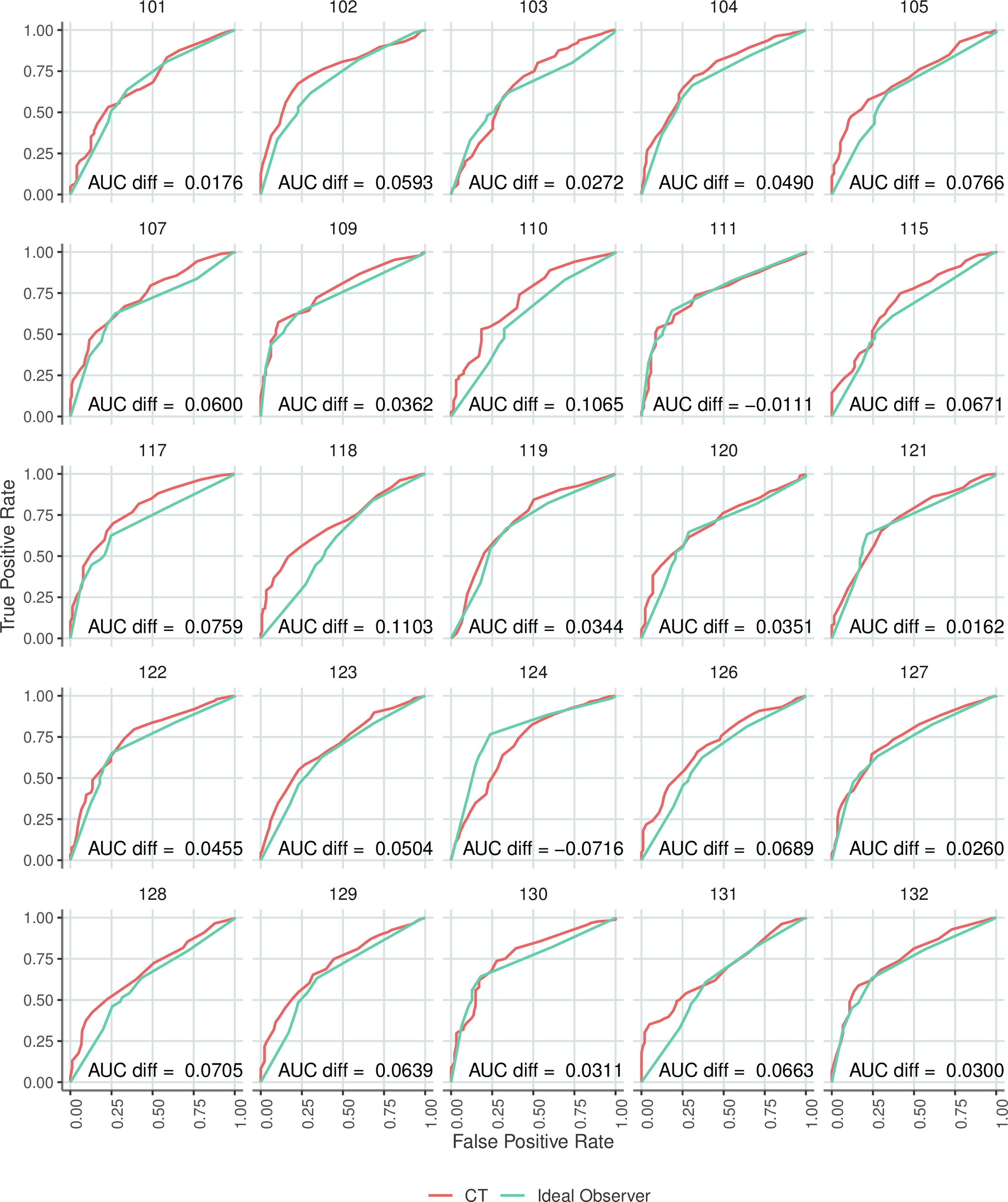

Supplement: S7 Fig — (TIF) [file pcbi.1010182.s009.tif]

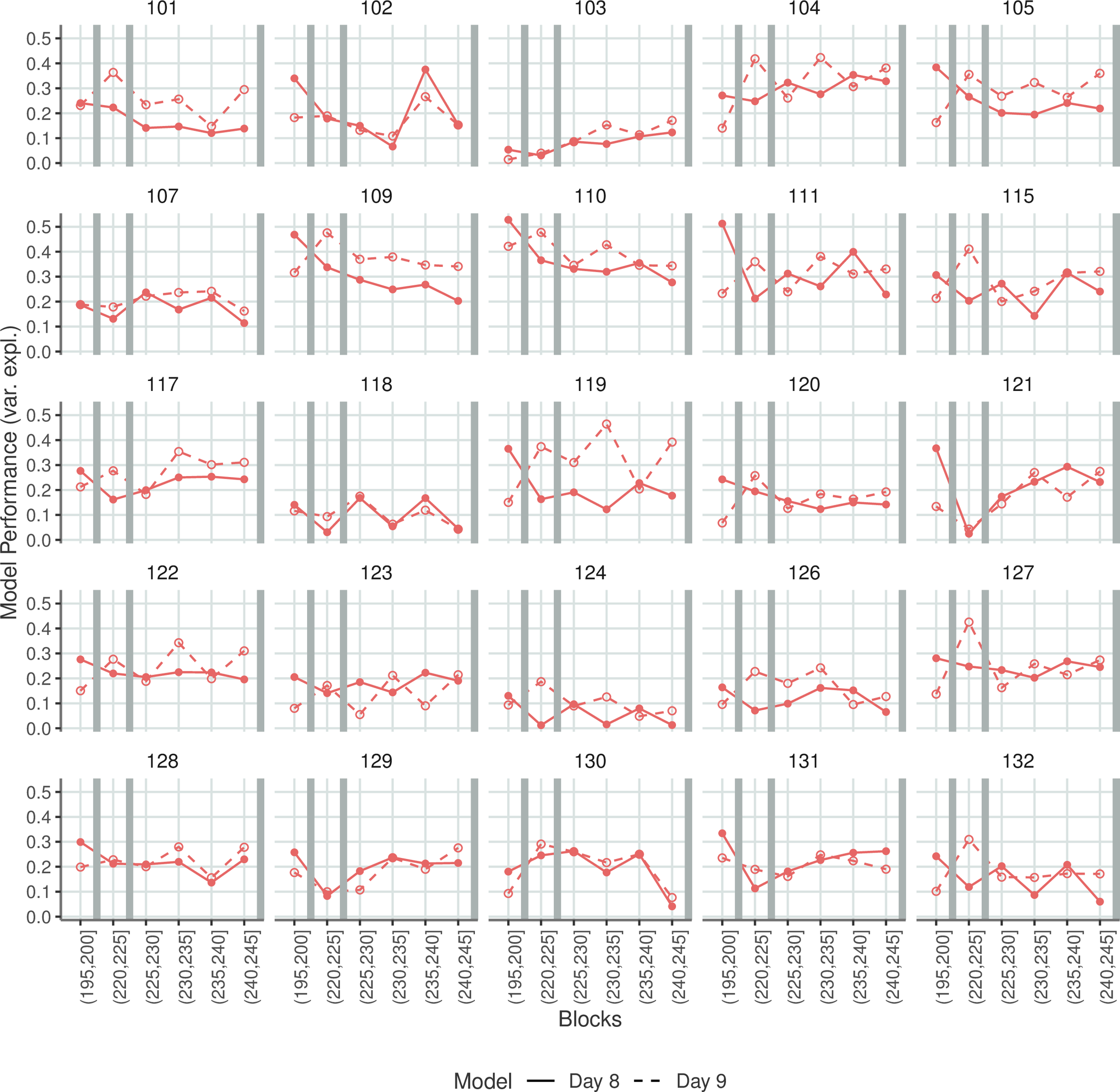

Supplement: S8 Fig — (TIF) [file pcbi.1010182.s010.tif]

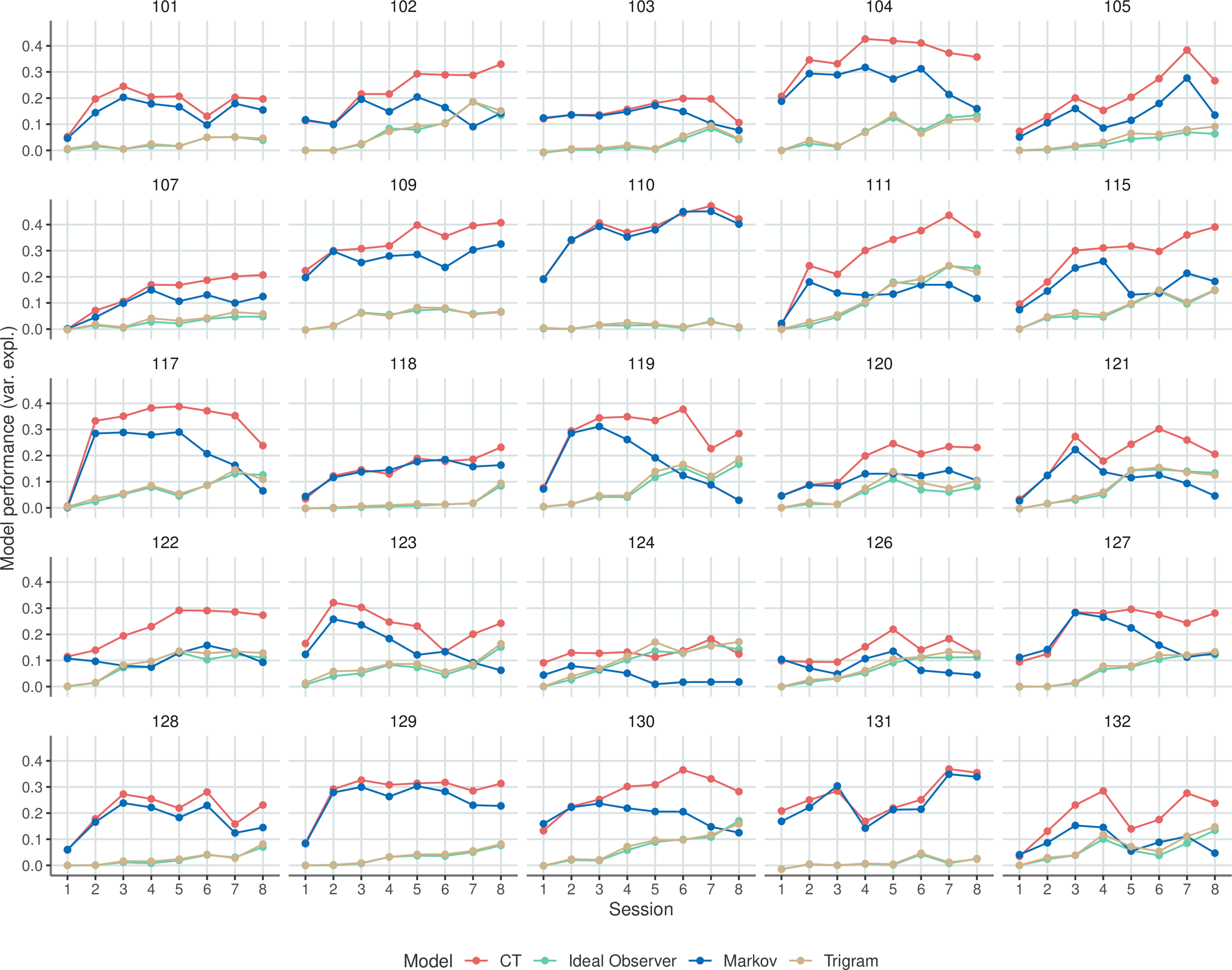

Supplement: S9 Fig — (TIF) [file pcbi.1010182.s011.tif]

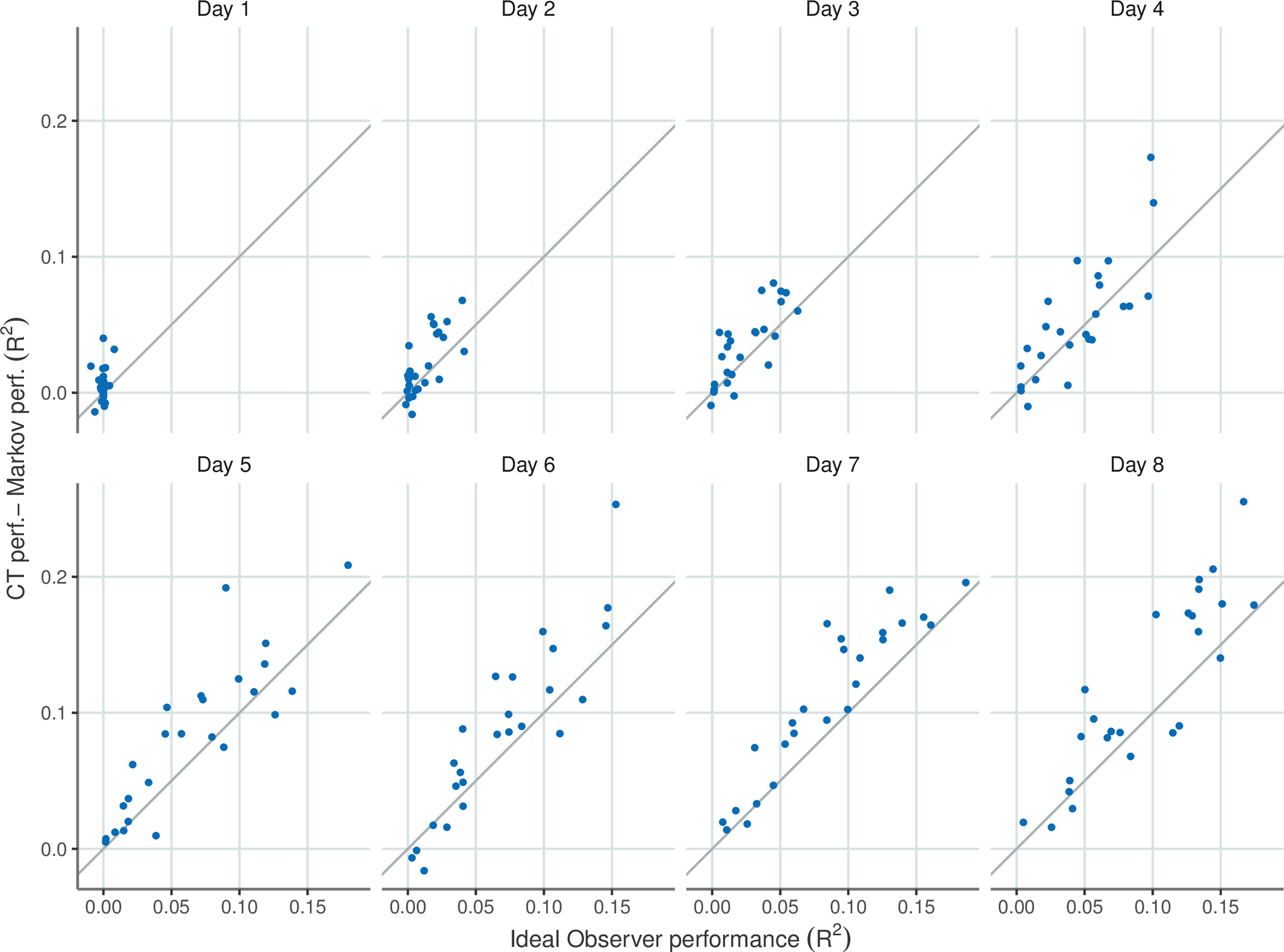

Supplement: S10 Fig — Dots indicate the performance of the models for different individuals. (TIF) [file pcbi.1010182.s012.tif]

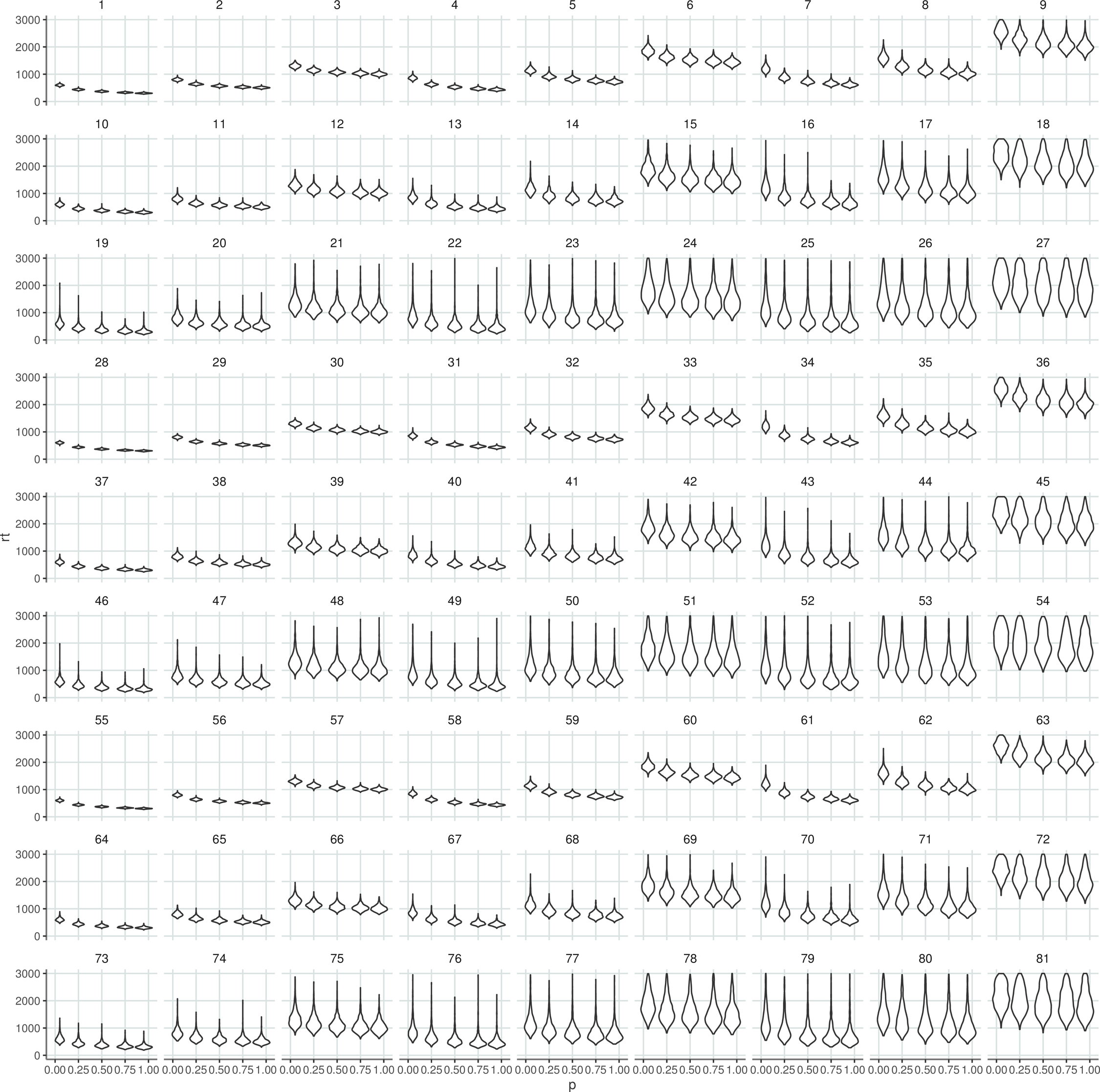

Supplement: S11 Fig — Each panel shows distributions with RT model parameters sampled from their respective priors. Distributions are shown as violin plots as a function of predictive probabilities. (TIF) [file pcbi.1010182.s013.tif]
