# Supplementary material for: Tracking the contribution of inductive bias to individualised internal models
Source: PLoS Comput Biol. 2022 Jun 22;18(6):e1010182. doi: 10.1371/journal.pcbi.1010182 (PMC9255757; doi:10.1371/journal.pcbi.1010182)
Supplement: Attachment — Submitted filename: R2R cogtom.pdf [file pcbi.1010182.s014.pdf]

We thank the Reviewers for their detailed and constructive criticism of the previous version of our manuscript. We believe we have managed to address all their comments appropriately and as a result the paper is now clearer. Below we give a detailed response to each individual comment (reviewer comments are in italics and our responses are in bold) with pointers to relevant changes in the manuscript (highlighted in blue there). As part of the revision to address the comments, we have added three new figure panels, two supplementary figures, one supplementary figure panel and two appendices.

Detailed response to reviewers

Reviewer #1: *Review of "Tracking the contribution of inductive bias to individualized internal models"*
Reviewer: Michael Landy

This is quite a fascinating paper, and I was quite impressed with the ability to infer internal models from reaction-time data alone. I think this paper is a substantial contribution to the literature and well worth publishing. I come to this paper with some background and my own work on sequential effects, but I was unfamiliar with the background on which the work was based (beamforming, iHMM's and how to infer them). As such, I think that this paper would benefit from a bit more of a clear tutorial and clarification, and the bulk of my comments below are about making the paper easier to read for the uninitiated.

We added a brief introduction on how hidden Markov models can be used for modelling and what advantage its infinite version has in Appendix A and added reference to it in Line 52 and Line 120.

Specifics (by line number, mostly):

Title: It's interesting that the title stresses inductive bias, whereas the bulk of the paper is about inferring internal models, and the bit about inductive bias, while quite interesting, only comes up at the very tails end of the Results section. I'd think a title that covers the rest would make more sense. The abstract also only talks about inductive bias in the final sentence, which is appropriate.

Thank you for highlighting this point. We agree with the reviewer that the narrative of the manuscript did not properly emphasise the contributions the study makes to understand the acquisition of an internal model and therefore we have updated it to provide the reader with the right perspective.

We believe that beyond providing a framework for flexible inference of high-dimensional models from reaction time data, it is an important contribution of the study that we can have valid insights into how the internal model can be partitioned into a component that is learned and a component that is readily available at the beginning of learning. We

believe that inductive biases that shape learning are little explored in the literature and will require more research. Still, in our setting the Markov model seemed to consistently account for the gap between the ideal observer and the model inferred by CT, which indicated that until participants aquire the right stimulus structure their behavior is governed by this simple model from day one of the experiments. We have rewritten the last paragraph of the introduction to better emphasise this point (lines 60–79) and we extended the discussion in this direction (lines 426-430).

Figure 2: The legend's description of 2C doesn't match the figure at all ("dots" versus what else? There are no left vs. right panels. What coloured labels are you referring to?).

Thank you for pointing this out. We corrected the caption of the figure and also corrected that the measure used for performance is not correlation but instead coefficient of determination.

155-157: This section (and probably some of the earlier text) leads the reader to think that you'll be studying the online learning of a model, and in particular, I thought at this point that you'd be applying the inference to multiple temporal sections of a session to watch that evolution. I only learned much later that your analysis treats the internal model as if it's stable and fixed within a session and, to the extent that you look at the dynamics of learned internal models, you do so at a much slower time scale (across sessions, i.e., across days). Reading the introduction and thinking about the task, I imagined there would be learning within a session and that you'd somehow track that with your inference method. So, I suggest clueing the reader in on what's coming earlier on in the manuscript, as I was disappointed when I learned that the fit of the model using 10 successive blocks was performed only once per session, and was validated by predicting EARLIER blocks.

That seemed particularly weird for session 1, when I would have thought the internal model wouldn't be at all stable during the early blocks, when there was little chance that the internal model would be at all stable. I'm surprised there was no discussion at all about within-session, trial-by-trial learning.

Thank you for pointing this out. We have included a paragraph in the first section of the results, which clarifies this point and provides arguments for this particular choice (lines 126–139) and we returned to this point in the discussion (lines 433–442) as well as in the Methods (lines 649-653).

176: "Participant-averaged performance of the ideal observer": At this point in the text, unless the reader goes off and reads the Methods carefully, it's not at all clear to the reader what the t-test is comparing and what is meant by performance. The phrase I've quoted would norrmally mean something about how well the ideal observer performs the task. But, by "performance" you mean how well the ideal observer correlates with the human RT data, i.e., the quality of its predictions. This should be rephrased and clarified at this point in the text.

Thank you for pointing out this potential confusion. We have clarified our notation and use the term predictive performance whenever we are referring to the ability of the *model* to predict subjects' reaction times for upcoming stimuli. Note that there is no explicit performance objective presented to the subjects, and therefore performance at the task is not unambiguously defined in this setting.

In addition, we have introduced a novel measure that can quantify in a principled way the ability of *participants* to use the contingencies in the stimuli to predict the upcoming stimulus. This measure is based on the measurement of KL-divergence between the subjective probabilities inferred from response times and probabilities characterising the ideal observer model. Thus, KL-divergence can be considered as a tool to quantify the performance of participants in acquiring the task statistics (see Fig. 6 G&H). This measure is also used to quantify the deviation between the inferred subjective probabilities and an arbitrary alternative model, such as the Markov model. We have extended the Methods section with a description of this measure (lines 836-841) and used it at the evaluation of individual internal models (lines 326-332, Fig. 6 G&H). Further, we justified this choice by an argument that establishes a normative link between the LATER model and KL divergence (Appendix B).

Table 1: I think it would be worthwhile to clarify where the numbers (5/8, 1/8) come from for the trigram model, i.e., half the time the trigram is applied beginning at a pattern trial and its prediction could be perfect, and half the time its aligned with a random trial and it can't predict at all. Not complicated, but worth pointing out explicitly.

The caption was updated according to the suggestion (Table 1 now turned into Fig. 3).

Figure 3: Violin plot: Is CT guaranteed to have better performance here (since it's more flexible), or is the plotted performance from the blocks that weren't fit (the earlier blocks in the session)?

The latter is correct: predictions are cross validated and therefore extra flexibility of the iHMM is not causing unfair advantage. The caption is updated accordingly (now Fig. 4.).

Also, in the legend, "mode" -> "model".

Corrected.

190: By "better internal model", do you mean closer to the actual generative model or do you mean closer to the flawed model the participant is actually using? The word "better" is best-suited to the former, while I think you mean the latter.

We meant the latter: the term 'better' qualified the ability of CT to reverse engineer the actual subjective internal model. Phrasing clarified (lines 225-226).

203: I like this analysis of the error trials. But, if you are interested in the predictions made by observers, why not run a version of the experiment in which some trials require prediction (i.e., the task switches from simple RT to prediction, where the task is to say in advance what color is coming up, then get feedback afterward on that trial)? That would be more informative and wouldn't require you to do the analysis only on rare error trials

We believe that errors provide an implicit measure of the predictions of the internal model and can therefore inform us about the deviations from the ground truth model. Note also, that the instruction participants receive about the task (that the sequence they see is random) avoids confirming that there is structure in the stimulus therefore a prediction task like the one proposed here would also alter other aspects of learning. We extended the discussion to include this point (lines 454–456).

228-229: It's not clear what these t-tests are testing/comparing (what the CIs are intervals of, especially since you don't say what the means are here). It's also unclear how participants notice an unsignalled change in sequence statistics fast enough for this to work, although of course it DOES work.

We believe this analysis is further evidence to the thesis that participants can hold multiple models and recruit them appropriately instead of constantly updating a single model. We now referenced multiple papers describing this theory and added a few sentences to clarify both the tests and what hypothesis we are referring to here (lines 260-269).

As I said, the whole manuscript treats internal models as if they are fixed and stable and, here, as if they are instantaneously swapped in and then stable. That's never discussed overtly nor justified, even though it seems, well, counterintuitive or almost certainly false.

We agree with the reviewer that under some circumstances (such as early during learning) our choice of inferring a single model cannot capture the full dynamics of learning. As we also mentioned earlier, fitting the model requires a large number of response times, hence this is a practical necessity. However, our analysis also highlighted that acquisition of stimulus statistics actually takes a number of days, which justifies investigating computational tools that do not aim for within-day mapping of the progression. We include this point in the updated discussion (lines 433-442).

Figure 4, and perhaps others: Minor point, but I read and review papers on my iPad (using iAnnotate) and a whole bunch of 4E, including all the data, was not visible. I'm guessing the figure has "layers" and they fooled my iPad's PDF viewer. In generally, you will likely want to flatten your figures (merge the layers) before final submission; that's safer. The legend states that stars mark significant differences, but I don't see any stars in the figure.

We have updated the figure and decided to remove the reference to stars. The comparisons are explicitly mentioned in text and therefore we decided to have a clearer figure by avoiding clutter on the figure itself.

237: There is no Suppl. Fig. S5B

Reference updated to Fig S7 (line 255).

267: S6 -> S8

Corrected.

Figure 5A: Shouldn't the ideal "outperform" the trigram model, because the trigram model can't infer the current phase (i.e., whether the current trial is a random or a pattern trial), whereas the trigram model can't make that distinction? That doesn't mean it will correlate better with human observers, but again it might be interesting to separately analyze those two types of trials. Yes, later on in the manuscript you do something about this distinction.

The reviewer is right that the ideal observer can infer the current phase whereas the trigram model cannot, however this does not translate to meaningfully better predictions of the response times. We refer the reviewer to Fig 7E. On this panel we plot our “Higher-order statistical learning” measure which relates to whether the participant can make a distinction between pattern and random trials. Two important features are: 1) this measurable distinction is really small even at the end of Day 8. 2) On the top two panels in Fig 7E, on Day 2, it can be seen that for some participants the distinction is done in the “wrong direction” meaning they react faster to random than pattern trials. This kind of structure in response times cannot be captured by the ideal observer model. We describe this notion in lines 378-380.

Figure 6C: What are the datapoints in the lower left? Extrema? Never mentioned in the legend.

The caption of the panel (Fig 7C in the updated manuscript) has been extended to include this information.

Legend Figure 6: "accounted for" in part B is about summing R^2 values, which you justify in the running text (as orthogonal elements). But, I thought model "performance" in these graphs was correlation (i.e., R, not R^2), so strictly they shouldn't sum. Please clarify. Also "advantage of normalized CT performance over the ideal observer model". Shouldn't that be over the ideal observer plus the Markov model?

On each plot performance is measured by R^2. We corrected the terminology in the caption of Fig 2 to clarify this issue. We chose to plot the predictive performance of CT normalised by the predictive performance of Markov against the predictive performance of IO because this measure emphasises the contribution of a learned model to the

internal model. Also, on Fig S9 this representation permits a direct visualisation of how the Markov gradually deviates from CT as learning progresses. We have clarified this at lines 354–357 and 360–362.

520: Is the starting phase of the sequence fixed or randomized across blocks? Across sessions?

For each session and each block the stimulus sequence started with 6 random trials and then the ASRT sequence with the same pattern element. This pattern element was randomised between subjects. During Day 9 and 10, when the sequence was changed, the first pattern element with which the block starts also (could) change, depending on what the randomised alternative sequence is. We also added a sentence at line 574.

537: The "correct" HMM is a ring of 8 states, half putting out a uniform distribution and half with deterministic output. You never state this explicitly, although it's intrinsic in your discussion of Figure 5F (which is an awesome figure, although I'm not sure how you pull a single inferred model from the inference, when the inference presumably provides a posterior across possible models).

Thank you for pointing this out. We have made it explicit that models shown in these panels are samples and predictive performance is an average over samples (current Fig 6D&F caption).

I'd think it would be worthwhile to point out that correct model.

We have included a description of the ideal observer model on lines 185-191 and added a panel to the figure that originally contained a table comparing the alternative models (current Fig. 3B, which was Table 1 in the original submission).

Even armed with that model, the ideal observer would start out with a distribution across states, and would only lock in after a few trials even if it knew the model with no model uncertainty.

That is correct and it is actually observable in data. However, we are only computing performance for the 80 real ASRT trials and its contribution to state uncertainty is miniscule after about 6 trials and hence it is not really influencing model performance.

544: \phi_{s,y} -> \phi_{S_t,y}

Corrected.

547: problem in 1 -> problem in Eq. 1

Corrected.

548-550: I found these two sentence obscure and even self-contradictory. Please clarify.

Text clarified (lines 615-617).

558: "uncertainty about the true model AND ACTUAL STATE OF THE STIMULI": I don't understand that latter phrase. In this paper, there is no visual uncertainty.

Clarified (line 625).

572 and nearby: I read the Methods early on just after starting to read Results. And, at this point in the Methods I assumed the pseudocode here was applied to groups of 10 blocks, then applied to a slightly later group of 10 blocks, and so on, to understand the dynamics within a session. It was only much later in my reading that I learned that it was only applied to a single, fixed stretch of 10 blocks per session. It would be good to clarify the approacher earlier.

We made sure to state that only a single model is fit per session both in the results (lines 126-139) and in the methods (lines 649-653).

588: Might be worth saying "countably infinite"

Updated.

590: Fig. ???

Corrected to Fig S2 (line 663).

608: "(e.g., one session)". First, this is the first time I learned that you assumed no learning was going on within a session (which is a bizarre assumption, although required to make this feasible). Second, the fitting is only for 10 blocks (40% of a session), so you don't really assume it's fixed for the whole session, although since you apply the estimated model to predict a different bit of the session, I guess you are assuming it's fixed for most of the session.

The reason for fitting the model on a subset of trials of a session is to have a cross-validated estimate on model performance. Our pilots showed that model performance saturated at using ten blocks for inference. We clarified this in the results section (lines 132-137) and in the Methods where we state using ten blocks for inference (lines 649-653)

Equation after 610: This bit of notation can be confusing. First, you switch mid-equation from event "S_t = s_t" to shorthand "s_t", even though they mean the same thing (I'd just use the latter). Second, there is a stray proportionalto symbol at the end of the first line. Third, the notation \hat{s}^t will, for most readers, make them assume this refers to a point estimate of the

state at time t. But, you seem to mean it to be an estimate of the probability that "s_t" is the state (given the current and past stimuli). That's a weird form of notation and will confuse people.

We edited the equation and added some more explanation to the notation.

611: "For filtering": Here I betray my outsider status: I'm not sure what "filtering" means in this community, so I was confused here. Also, it's worth reminding the reader what is meant by "even if responses times are not considered...". What you mean is that they aren't used in the performance metrics, but are used for model fitting, right?

We have clarified the text (line 678).

Table 2: The column headers here are completely messed up, so I couldn't really check/parse this table. Also, what are all they "[1]"s supposed to mean? Looks like some latex formatting got into the table itself.

Thank you for noting this, we have corrected the format.

616-619: Here, at last, is where you finally explicitly state what you are doing about the dynamics, without previewing earlier or justifying that this is a sensible thing to do.

See above our responses to the related points.

Table 3: This table also has similar messed-up formatting to Table 2. Is \alpha_0 the same as \alpha? Where is \gamma used? What is the "\cdot \varepsilon" doing in the definition of \hat{\pi}?

Thank you again for highlighting this, we have corrected the format.

631-635: I do STAN model-fitting in my lab, but you are clearly doing something fancier than I've done, so I can't really parse this paragraph (and am unfamiliar with NUTS ;^)

The combination of Hamilton Monte Carlo with slice sampling extends the capabilities of STAN. The NUTS, a.k.a. no-U-turn sampling is a built-in method of STAN that can be seamlessly invoked for better mixing.

Equation after 642: Why are you using capital letters for \phi and y all of a sudden?

Corrected.

Eq. after 659: Yes, I think this is basically the LATER formulation (I've used it in one paper, but didn't go back and check). The one weird thing about this formulation is that theoretically an outlier value of r_n could be negative, which would be a problem. For that matter, it could even be zero or very small (i.e., huge tails on the RT distribution).

We clarified this question in the update: we used a truncated Normal distribution to avoid such cases (line 732).

679: Running it on a simulated observer is obviously a good thing to do. But, did the analysis recover the model that the simulated observer was using? How would you know? How would you score how well it did at recovering the internal model?

Thank you for noting this. We chose to measure the efficiency of CT via matching the ground truth prediction probabilities and the reverse engineered predictive probabilities because this is the sole objective learning is optimised to. We clarified this at lines 738-742. We have also added a new panel, Fig S3B that shows that the recovered model does just as well in response time prediction as the actual internal model of the synthetic participant.

686: "below the threshold...": What was that threshold and how was it determined?

We have clarified the sentence in Line 765. Our goal with Fig S3D was to establish that humans fall within the range where our inference method was validated.

733: It's only in this line that you talk about explicitly the issue of models changing under the hood in early trials of a session.

See our replies and references to the updates above.

742 et seq.: I was confused by this section and by the corrresponding Results text. First, in this section it wasn't clear what was meant by an error (a wrong button press, of course). It would have been nice to clarify up front that here would would ask whether such finger errors relate to the posterior probabilities of each possible stimulus, both in terms of missing the correct button because it's less likely, and in terms of which button you hit instead, because its probability is high. The main Results text didn't make this clear either.

Thank you for the suggestion. We have updated both the results (lines 233-237) and the methods (lines 822-825).

Finally, the ROC idea really is kind of nonsensical, i.e., it doesn't relate clearly as a process model of how finger errors are made.

Our intention with the ROC was not to suggest a process for error generation. Instead, ROC was used as a tool that is well motivated by information theory to characterise the level of advantage the CT model has over the Markov model. The plots in C should be complementary to the bars in A and B but instead of showing a single statistic (accuracy), it shows that CT is in fact a better predictor overall.

S2A: The Table in the lower-left of this panel might as well have actual Greek letters as the row headings ;^)

Corrected.

S2B: Again, the labels on the axes should be explained. Is this an R or an R^2? In the legend description of A it says "symbols with different colors and shapes", but this is in panel B, not A.

Similar to other parts of the manuscript, performance is measured in R^2. We have clarified this in the updated caption (S3C) and also corrected the misplaced sentences.

S6: Are there 64 points in each plot corresponding to the different trigrams?

The reviewer is right (except that we omit some of the 64 if there are not enough data points), we have clarified this point in the caption.

Reviewer #2:
The authors investigated an implicit visuomotor sequence learning task and developed a computational method to reverse-engineer participants' internal models of the serial dependence through their reaction times (RTs). One major novelty of their method was the use of the infinite hidden Markov model (iHMM) to capture the potentially infinite space of serial patterns that participants might acquire. The authors found that, in explaining participants' variation in response times, this iHMM model (which was called the CT model in the paper) outperformed both the ideal observer model that follows the ground-truth transition rules and a few Markov models that only tracks the transitions between observable states. The explaining powers of different models changed over the training process, with the earlier stage better approximated by a first-order Markov model and the later stages better by the ideal observer model and a second-order Markov model (i.e., the trigram model). The authors concluded that the failure of achieving an internal model of ground truth as well as its individual differences resulted from specific inductive biases, in particular, a prior belief in first-order Markov transitions.

I think the application of iHMM to modeling human participants' internal models is novel and insightful. The work is also technically solid. The writing is overall elegant and clear.

We thank the reviewer for the accurate summary and the kind words.

But I also have some concerns about the major conclusions of the paper, which I shall specify below.

Major concerns:

1. What parameters of the CT model characterize individual participants' inductive biases (prior beliefs)? In the CT model, participants were assumed to update their prior beliefs from time to

time in a Bayesian way. The deviation of their behaviors from the ideal observer's depends on their priors. Before reading through the paper, I had thought it would be the hyper-parameters that differed between different individual participants. But when I came to the Methods section, I found the same set of hyper-parameters were used for modeling all participants' internal models. Then what contributes to the individual differences in the learned internal models (e.g., Fig. 5F)? Did the individual differences just reflect some random variations in participants' Bayesian inference? Or, did I miss anything?

We did not seek to identify the inductive bias by searching through the space of possible priors directly. We reverse engineered each participant's internal model based on their reaction times, and these internal models are hypothesised to be a result of the combination of the individual's inductive biases and a learned component that reflects the properties of the stimulus sequence. Therefore, the information regarding each individual's inductive biases lies in the inferred CT model parameters. Instead of extracting these priors directly, we used an Ansatz to account for the deviation between the ideal observer and CT models. We found that the Ansatz (i.e. the Markov model), that was motivated by offering a parsimonious explanation for temporal dependencies, could account for the overwhelming portion of the deviations. We have clarified this point at lines 285–294. We also inserted pointers to the newly introduced Figure 3B such that the inferred internal model can be directly compared to the graphical representation of the Markov model. The inductive biases can be richer than the Markov model and it might be a subject of further investigations to account for an even larger portion of the deviation, and this question opens up novel avenues towards transfer learning. We discuss this point in the discussion section (lines 426–430).

2. The authors had concluded that the (first-order) Markov model is part of participants' inductive biases. I was wondering how this conclusion could be compatible with the best-fitting model—the CT model (i.e., iHMM internal model). Links should be made between the internal model predicted by the CT model at early training stages and those of the Markov model. For example, Markov-like internal models might be the emergent properties of iHMM after limited learning experience. Moreover, if so, could it still be claimed that the Markov model constitutes participants' inductive biases?

The iHMM is a very expressive class of models that contains the Markov model as a special case. Consequently, when using CT to map the internal model in the early stages of learning, if the internal model is indeed a simple fully observed Markov model, the inferred iHMM should be the same. Fig S9 shows that the predictive performance of the CT models on the earlier days are very close to the Markov model for each subject, suggesting that CT model captures the regualirities encoded in a Markov model. In order to provide a more direct comparison between the early CT model and the Markov model, we added Fig 6G which shows the average KL divergence between the predictions of the Markov model and the inferred iHMM on day 2. We have clarified this at lines 302–304. Note also that in line with this inductive bias interpretation, Fig S10 suggests that a

divergence between the predictions of CT and Markov appears and grows as the internal model moves toward the ideal observer model.

*3. The authors had shown that the trigram model, a model with the second-order serial dependence, could not explain specific features in participants' RTs (Fig. 6E). But how about a "quadgram" model with the third-order serial dependence? Considering that it only involves 4*4*4*4 = 256 possibilities, while there were 85 trials/block * 25 blocks = 2125 trials per session and a total of 8 training sessions, it is not a crazy idea that participants might acquire the third-order serial dependence. Besides, such quadgram model seems to be more computationally tractable than iHMM.*

It is a valid point that the trigram model could be extended and arbitrary n-grams could be considered. However, we claim that regardless of the size of such n-grams, the iHMM will pick up on the length of temporal dependencies that determine the responses of individuals. We demonstrate this idea when showing that Markov and Trigram models together achieve the performance of that of the iHMM. Our data shows that as long as the trigram model cannot predict the response times of an individual, the iHMM shows predictive performance that is indistinguishable from the predictive performance of the Markov model (Fig S10). This shows that iHMM extends state space when necessary but results in a simpler model when there is no evidence against this simpler model.

4. Could there be any model-free plots and descriptions of the RT results?

We have added a supplementary figure (S5) with the overall RT distributions and how well they are captured by the different models.

5. Working memory capacity had been measured for each participant. Did it correlate with participants' task performances or their internal models?

We did correlate our working memory measures with task-performance evaluated using the newly introduced KL-divergence between the recovered internal model and the generative model of the task but there was no significant correlation between them.

Minor issues:

1. I agree that "Cognitive Tomography" (CT) is a cool term. However, I do not think the "CT model" is an appropriate term for the specific CT model based on iHMM, because all the other models in the paper share the same CT framework (internal model + response model). Something like the "iHMM model" might be better.

We agree with the reviewer that technically the main difference between the ideal observer model and the CT model is that one uses a fixed-parametrization HMM while the other is using a non-parametric extension. Nevertheless there is a conceptual difference as well, which is worth emphasising with the terminology: while the ideal observer

assumes that the parameters of the graphical model is determined by the environment, cognitive tomography attempts to determine both model structure and model parameters based on data alone without reference to the stimulus statistics. We believe that the choice of pitting HMM against iHMM is less helpful for the readers to conceptualise the key differences between the alternative models. We emphasise the key distinction in the part of the results that presents the alternative models (lines 185-191).

2. Lines 208–214 and Figure 4A: It seems meaningless to compare the model-predicted proportion of top rank to the chance level. If one model has an overall higher accuracy to predict the incoming stimulus than the other model, its proportion of top rank would be naturally higher than the latter for both correct and incorrect responses. What matters is the discriminability of the proportion of top rank between correct and incorrect responses, such as the ROC reported in Figure 4C.

The reason we chose to use the top rank is because it shows that if we take the maximum a posteriori prediction of a model, then the ideal observer will systematically misjudge which button will be pressed.

Line 212: "However, it also assigned the highest probability to the upcoming stimulus in incorrect trials (0.315, n = 2777, p = 1). " The statistics in the parentheses do not seem to support the statement.

Thank you for pointing this out. The statistical test (now in Line 251) was incorrectly done in the other direction, we have now corrected it.

3. I could not quite understand what Figure 4E could tell us. It seems that every model (not necessarily the CT model) could have better predictions for participants' behaviors when the test statistics were more similar to the training statistics.
I felt even puzzled when I came to Line 616, which reads "throughout the execution of the task, the internal model of the participants is continually updating." If the updating modeled by the CT model was close to participants' actual updating, shouldn't we see similar model performance (R^2) in different test sessions, no matter whether the test statistics were similar to the training statistics or not?

We included figure 4E because we believe it shows that instead of having a single model and updating it, people realise there is a distinct pattern to be learned and recruit a different model during those periods. And the alternation of how well these models predict the statistics at hand shows that they are actually recruiting the relevant model and only updating that model. We added a paragraph on this idea into the main text (lines 260-269).

4. Some important details about modeling fitting and comparison methods should be made more explicit in the main text. For example, (1) whether each session of each participant was

fitted separately, (2) whether cross-validation was used, and (3) for cross-validation, which parts of data were used as the training set and which as the test set.

We have updated the text to better emphasize the design of analysis (lines 133–137 and 150–152). For additional details also see section Train and Test Datasets of the Methods.

Some of these details seem to be described in Table 2, but Table 2 is mis-placed in format and hard to follow.

Corrected

Line 565: "ten consecutive blocks of trials". Why ten blocks? Weren't there 25 blocks in each session?

We added a clarification to why we chose to use 10 blocks for training the model (lines 650–652).

Line 572: "For each of the 60 posterior model samples". What does the "60" mean?

Thank you for highlighting this. We have removed the reference to this number since it is given at a later part of the text (line 702).

5. Line 567: "response times smaller than 180 msecs in each block removed". What percent of trials were removed?

We added the percentage of trials removed in lines 639–640.

6. Line 7: "intuitive psychology" seems to be rarely used in the literature. The term "folk psychology" is more common.

While these terms have closely related meanings and are sometimes used interchangeably, we believe the term intuitive psychology describes the notion we are referring to slightly better. In our understanding, folk psychology is often associated with verbally expressible concepts and descriptions that people use to communicate regarding human behaviour, whereas we also want to emphasise the implicit, nondeclarative knowledge contained in subjects' internal models.

7. Finding participants' internal models to deviate from an ideal observer does not seem to be new or surprising. Why were there so many figures devoted to the comparison between the CT model and the ideal observer model? A comparison between all the models (such as Fig. 5) is more informative and might be better to be described earlier in the paper.

Indeed, there are reports available on the deviation of humans from the ideal observer model. CT promises us to take a constructive approach by reverse engineering the actual

internal model. In order to show that it lives up to its promise we needed to demonstrate that it is indeed the internal model that we are inferring, and once we do this we can characterise *how* the internal model deviates from the ideal observer.

We agree with the reviewer that comparison with multiple models provides better insights. However, before showing alternative modes, with Fig 1-5 we intend to introduce our approach and establish its validity. Only once we have shown evidence that our approach recovered meaningful internal models can we make conclusions about the internal models it suggests. For this reason, the ideal observer model is used as a baseline to gauge the CT model's performance on various prediction tasks (e.g. that CT generalises to predicting erroneous key presses). Fig 4, in addition to showing that the learned internal model is different from the ideal observer, also intends to convey that stimulus statistics is steadily being acquired over the course of the eight days of the experiment.

8. Could there be a graphical illustration for the ideal observer model, similar to Fig. 5F? Maybe by enhancing Table 1.

Thank you for the great suggestion. We have included a panel on the proposed figure (now Fig. 3).

9. The legends of Fig 5D–5F are a little confusing. When I first read “Participant 102 finds a partially accurate model by Day 2 (D) and a model close to the true model by Day 8 (F)”, I had thought (D) and (F) were only about Participant 102.

We have clarified the caption.

10. Typos:
Line 221: a space is missing between “model” and “as”.
Corrected
Line 590: The figure number in the parentheses is missing.
Corrected
Table 2: The headings of the table seem to be mis-placed.
Corrected

Reviewer #3:
The manuscript presents a new method for inferring subjects' internal models from response times in a sequence learning task. Because the task's statistical structure is unknown to the subjects, the assumption that the subject's internal model matches the true generative model of the task (the ideal observer assumption) does not hold. Thus, the authors use a flexible class of dynamical models (iHMM) to represent subjects' internal models and combine it with a behavioral model linking subjective probabilities to response times. The iHMM-based internal models estimated from response time data are shown to predict response times better compared to the ideal observer. By considering an alternative model, which assumes no hidden

structure but only Markovian dependencies, the authors show that all subjects start with a bias towards simple temporal dependencies, but some subjects learn a model closer to the ideal observer.

The conceptual introduction to the problem is very clear. Particularly, the explicit distinction between the subject's internal model and the behavioral model accompanied by the graphical model notation (Fig. S2) is quite helpful. The results are interesting and the "cognitive tomography" method constitutes a relevant contribution to the recent literature on inferring internal models from behavioral data.

We thank the reviewer for the summary and for the encouraging words.

However, the description of the methods could be improved in terms of clarity and level of detail and some aspects of the results need clarifying statements or additional analyses:

- Clarifying what exactly constitutes a state of the dynamical system in the article's main text would help readers not well-versed in HMMs and similar models.

We added a brief introduction to HMM and iHMM (and reference in Line 52 and Line 120) with an example that helps understand the different parts of a hidden markov model and why its infinite extension may be useful (Appendix A).

Relatedly, while the graphical notation for the model (Fig. 2A) is very informative once understood, it is worth a little more explanation: e.g., the authors could show how the true dynamics of the task (i.e. the ideal observer's model) or an instance of the Markov model look in the graphical notation, which would make it easier for the reader to appreciate the inferred models (Fig. 5D,F).

Thank you for the great suggestion. We have extended Table 1 of the original paper and transformed it into Fig 3, which now features two graphical models, one corresponding to the ideal observer, the other to a Markov model.

- The validation of the inference method on synthetic datasets is a bit scarce. For a model of this complexity and a highly customized inference procedure, I would expect to see some prior predictive checks, inference diagnostics (e.g. r_hat, effective sample size), and posterior predictive checks. As a guideline for reporting Bayesian analyses, I suggest Kruschke (2021).

Thank you for referring us to this. Here we will go through the different proposals one by one.

Prior predictive checks. In order to demonstrate synthetic response time distributions in our synthetic experiment, we added Fig. S11.

Inference diagnostics. 1, In order to test the assumption about the variance in reaction times, we calculated residuals of response times for each participant individually. Theoretical prediction of the distribution is tested against the actual residuals on Fig. S4. We clarified this point in lines 156–159. 2, We also stated that we selected 10 consecutive blocks of trials for inference because that is where model prediction performance saturated (lines 649-651).

Posterior predictive check. We added a supplementary figure showing marginal distributions of response times for three models: CT, ideal observer, and Markov model (lines 164–166, Fig. S5).

- The evaluation presented in Fig. S3B suggests that the response time prediction performance is not particularly good. Even for synthetic datasets with response time standard deviations comparable to real data (the darker dots), the R^2 values are mostly between 0.25 and 0.75. Is this just the result of the inherent variability in the response times due to the LATER model (which the better performance in predicting subjective probabilities might suggest) or is this due to a failure of the inference method? Could one compare the response time prediction performance against an upper bound on the response time prediction performance computed from the ground truth synthetic internal model? How well are the parameters of the response time model (tau, mu, sigma) recovered by the inference method?

When conditioning on the internal model, the subjective probabilities will be deterministic, while the response times will not be. Therefore, if we knew the exact internal model, there would be no variance in subjective probability prediction, while there still would be uncertainty in response time prediction due to its inherent variability.

We thank the reviewer for suggesting an interesting comparison, we added panel Fig S3B that shows that the recovered model does just as well in predicting response times as the actual internal model of the synthetic participant.

- The model comparison based on R^2 is not really convincing, because it does not take into account the significantly higher model complexity of the iHMM-based model. The authors chose R^2 because not all models are Bayesian. To my understanding, the only non-Bayesian model is the trigram model, which is not central to the argument in the paper, while the ideal observer, the iHMM-based model, and the Markov model are Bayesian. If this is correct, the authors should perform a Bayesian model comparison for these three models. If this is incorrect, please expand the description of the models in the paper to make clearer how they are fit to the data.

Good point, there is an order of magnitude more complexity in the iHMM model. That is why we opted to only consider performance metrics on a test dataset not overlapping with the training datasets. Therefore only those complex models can prevail that have sufficient generalisation capabilities. A model that overfits the training data could not achieve superior performance on test datasets.

- In the Section "Trade-off between ideal observer and Markov model contributions", the inferred internal model is only compared quite indirectly to the ideal observer model, via their predictive accuracy for response times. Is there a more direct way to assess the distance between an iHMM and the true HMM employed in the experiment? The evaluations presented in the original iHMM paper by Gael et al. (2008) suggest that there is.

In order to present a more direct measure of how the internal model is related to the ideal observer model, we introduced a new measure. We computed the KL-divergence between the predictions of the inferred internal model and the true generative probabilities of the task. We show how this measure changes as participants acquire knowledge about the task (Fig 6 G&H).

Apart from KL-divergence being a standard distance measure for probability distributions, this quantity also constitutes an objective measure of participants' task performance (objective in the sense that it is independent of the mean and scale of their response times and only considers how well they learned the true probabilities). We added an Appendix to describe a normative interpretation of the LATER model, which leads to KL-divergence if we assume that participants' goal is to minimize the expectation of their response times (Appendix B).

- I agree with the point made in the discussion, that POMDPs are a possible alternative formalization for the problem at hand. While the authors acknowledge in the introduction that the internal model of the agent need not be identical to the true generative model of the task, this point also applies here: An agent might assume that their actions influence the state, while it is actually not the case in the true generative model. Furthermore, employing a POMDP formulation might also shed light on internal costs relevant to the task (e.g. computational costs), which are absent from HMMs without explicit modeling of actions. I think these points are worth further discussion.

Thank you for coining this. We have extended the discussion with a reference to this idea (lines 462–464).

Minor points:

- The argument in the introduction for moving beyond ideal observers could be strengthened further by including relevant literature making similar arguments (e.g. Feldman, 2013, Beck et al., 2012).
We have added these references to the introduction.
- "learning a novel statistics" should be "learning novel statistics" (p. 3, l. 22), same on p. 4, l. 58, p. 10 l. 200, 201, 222
Corrected.
- Fig. S3A refers to Gael (2011). Should this be Gael et al (2008) as in the caption and in the bibliography or is it referring to a different paper?
Corrected.

- "and we formulated as a trigram model" (grammar and meaning??)
We clarified the sentence (line 199).
- Fig. 5B is not referenced
Corrected
- Latex: use $M_\text{education}$ (\text environment for whole words) instead of $M_{education}$
Corrected.
- p. 22 l. 590: missing figure reference
Corrected.
- Table 2: headings seem to be broken
Corrected.
- p. 14: spelling of normalized / normalised is inconsistent
Corrected.

Have the authors made all data and (if applicable) computational code underlying the findings in their manuscript fully available?
The PLOS Data policy requires authors to make all data and code underlying the findings described in their manuscript fully available without restriction, with rare exception (please refer to the Data Availability Statement in the manuscript PDF file). The data and code should be provided as part of the manuscript or its supporting information, or deposited to a public repository. For example, in addition to summary statistics, the data points behind means, medians and variance measures should be available. If there are restrictions on publicly sharing data or code —e.g. participant privacy or use of data from a third party—those must be specified.

Reviewer #1: Yes

Reviewer #2: None

Reviewer #3: No: While zip files containing data and code were available, the passwords for these files were only available upon request from the authors.

We apologise for omitting this. We provide these details below:

Data:
https://www.btorok.me/cogtom/cogtom_data_bt.zip
Password:
iC2rm!k39#A%q4YwJ3qx

Code:
https://www.btorok.me/cogtom/cogtom_code_bt.zip
Password
4#fC!9D5M#vGXJ$m1LV2
