# Supplementary material for: Tracking the contribution of inductive bias to individualised internal models
Source: PLoS Comput Biol. 2022 Jun 22;18(6):e1010182. doi: 10.1371/journal.pcbi.1010182 (PMC9255757; doi:10.1371/journal.pcbi.1010182)
Supplement: Attachment — Submitted filename: r2r cogtom revision 2.pdf [file pcbi.1010182.s015.pdf]

Revision 2

I was impressed with this paper the first time, but needed a bit more clarity in the presentation. This version improves that quite a bit and adequately responds to my and the other reviewers' requests (IMHO). My comments are pretty minor.

Specifics:

lines 99-100: This says that sessions were on consecutive days, but the Procedure says they were spaced a week apart.

It was indeed weekly spaced, thank you for spotting the error in the text. Updated.

Figure 3: The diagram on the lower right is probably supposed to be the Markove model, but it is neither mentioned nor described at all in the legend.

Corrected.

276-277: "successfully recruit previously learned models": I thought this referred to the fact that on Day 10 the better-performing model tracks the switching. That's clear in Fig. 5E, but is not exactly convincing across subjects glancing as Fig. S8 and no summary across subjects is provided.

Text and caption updated.

294: vanes -> wanes

Corrected.

Fig. 6D,F: The correspondence between the model samples and participants/days is not clarified in the legend and is indicated by red circles on day numbers and a skinny red line connecting the model to that day that I missed completely until I stared at the figure for quite some time. You should make the connections more obvious AND mention it in the legend. Another option (not mutually exclusive) is to put a title in the corner of each model sample that says something like "Participant 102, Day 2".

The caption has been rewritten.

327: The main text here only suggests that KL is used to measure the match between predictions, whereas the Appendix gives another justification for why KL is the right thing to do. You should allude to that other justification here as well.

Text updated.

338: identified FOR all?

Corrected.

340: FOR several participants (or maybe IN, but not AT)

Corrected.

436: characteristic OF all participants

Corrected.

3 after 603: A glitch here. S_t,Y_t \memberof \mathcal{N}. The sequence... [Yeah, I know my latex is wrong ;^)]

Corrected.

695: inbetween -> in between

Corrected.

697: last 60 -> the last 60

Corrected.

Table 1, Hierarchical prior over state transitions, Notation: Shouldn't that be \alpha,\gamma ???

Corrected.

711: predictions many -> prediction of many

Corrected.

759: seen on -> seen in

Corrected.

775: are subset -> are a subset

Corrected.

822: take THE mean ... over THE last 60

Corrected.

824: IN Fig. 5

Corrected.

827: predicting A correct trial

Corrected.

Supplement, p. 2, para. 2: The reference for Hierarchical Dirichlet didn't get filled in.

Corrected.

Supplement, Fig. S3: I have a notation here "Compare the resulting HMMs". I can't remember what sort of comparison I was thinking of... ;^(

We did a direct comparison of the ground truth synthetic internal models and the recovered internal models through calculating KLs between inferred and ground truth predictive probabilities. However, we decided to omit the figure from the manuscript since the extensive explanation it required diverts attention unnecessarily.

Appendix B, para. 2: Note that THE quantity we obtained…

Corrected.

Reviewer #2: I have no further questions.

Reviewer #3: I appreciate the effort the authors have made to address my previous comments and I think the clarity of the manuscript is improved. Specifically, the description of the iHMM is now more detailed and accessible for uninitiated audiences. My concerns about the model validation and comparison have been resolved by the addition of the new version of Fig. S3B and the clarified description of how the models were fitted and evaluated. With most methodological concerns out of the way, I only have one remaining minor issue: In my previous review, I asked for MCMC diagnostics because of the custom inference method and high model complexity. While the authors have pointed to a plot validating an assumption of the model and clarified how much data were used to fit the model, they have not shown evidence for the convergence of the MCMC method.

To check proper mixing of the MCMC chains, we checked whether marginal distributions of the response time parameters as well as the number of states represented were identical across chains.

Our analysis showed reliable mixing of Sigma, mu, tau0: across chain variance was substantially lower than that within the chain. Occasionally in some of the chains, the number of states got stuck in one of the modes but in most of the cases this singular K was inside the support of the other chains.

All results presented were marginalised over all chains, therefore the effect of the exceptional singular chains were also annulled.

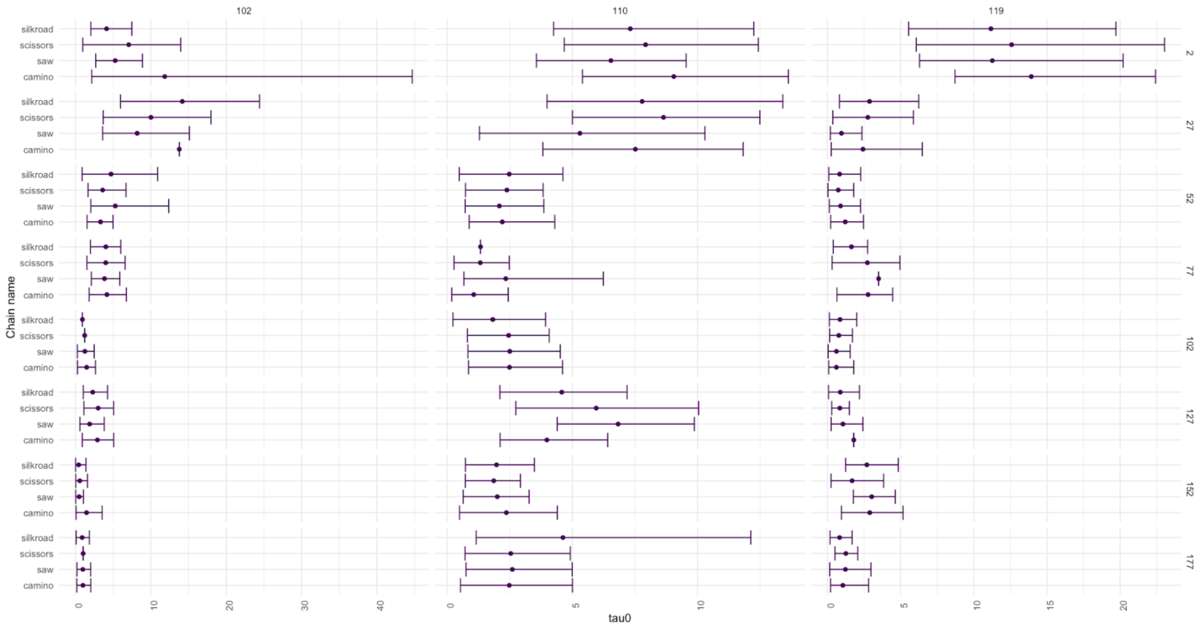

102
110
119
silkroad
scissors
saw
camino
Chain name
2
27
52
77
102
127
152
177
0
10
20
30
40
0
5
10
0
5
10
15
20
tau0

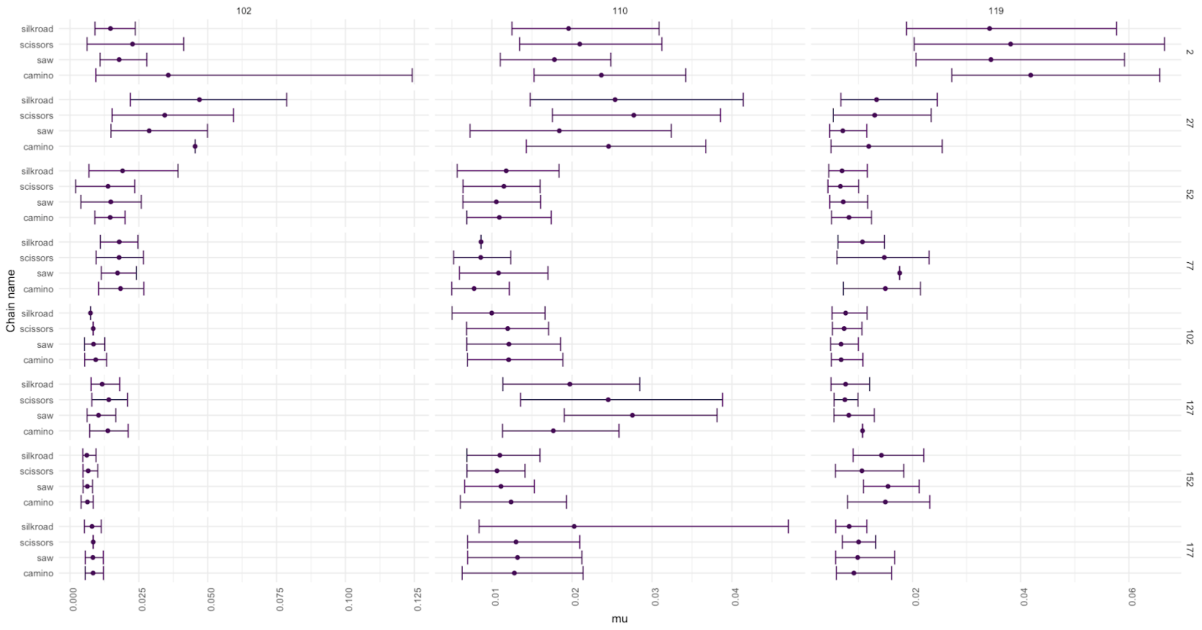
102
110
119
2
27
52
77
102
127
152
177
silkroad
scissors
saw
camino
Chain name
0.000
0.025
0.050
0.075
0.100
0.125
0.01
0.02
0.03
0.04
0.02
0.04
0.06
mu

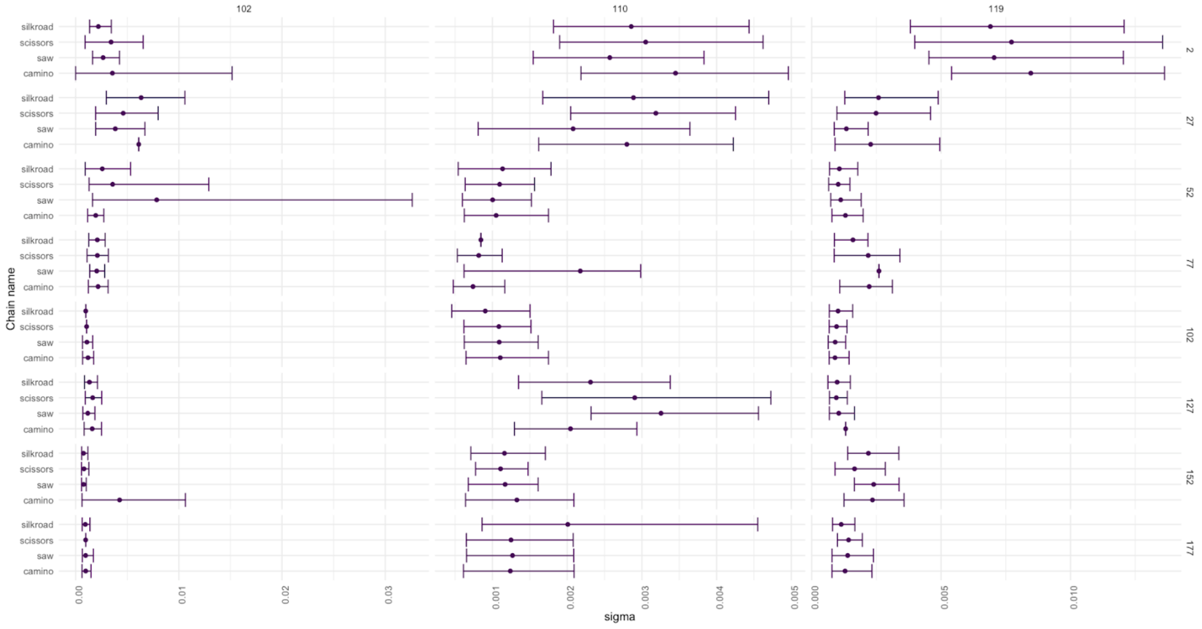
102
110
119
2
27
52
77
102
127
152
177
silkroad
scissors
saw
camino
Chain name
0.00
0.01
0.02
0.03
0.001
0.002
0.003
0.004
0.005
0.000
0.005
0.010
sigma

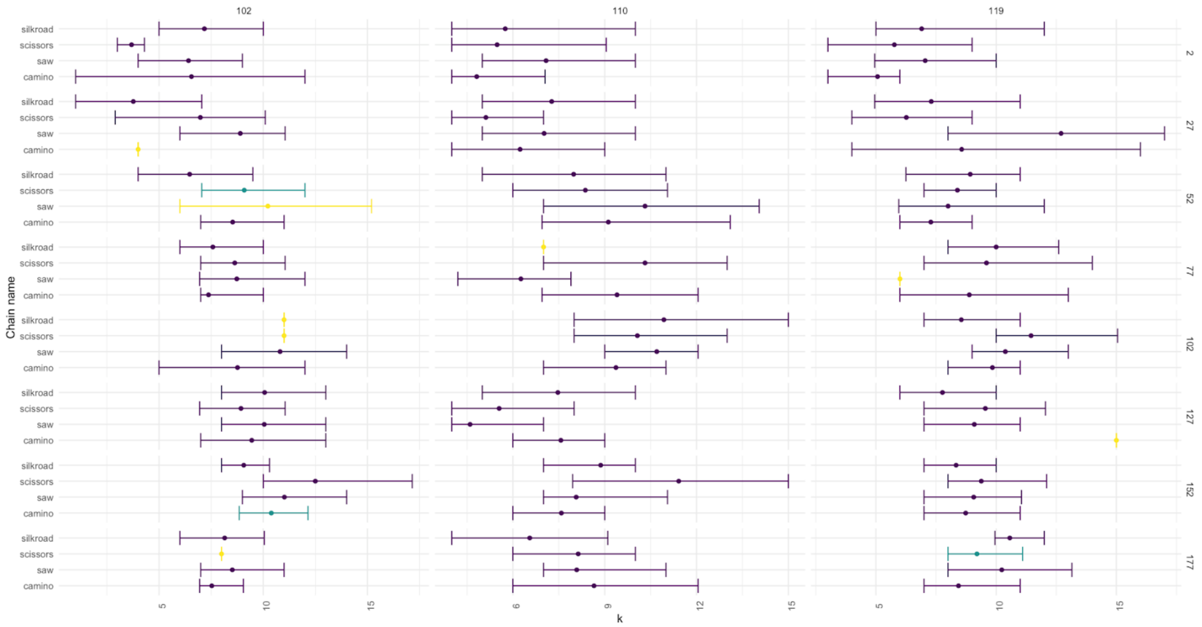

Further, we would like to mention that we noticed a minor mistake on plot Fig5C where the AUC difference numbers were incorrectly shown. These are now corrected. Similarly, we replaced the same type of figure (Fig S7) with the corrected numbers.

Have the authors made all data and (if applicable) computational code underlying the findings in their manuscript fully available?

The PLOS Data policy requires authors to make all data and code underlying the findings described in their manuscript fully available without restriction, with rare exception (please refer to the Data Availability Statement in the manuscript PDF file). The data and code should be provided as part of the manuscript or its supporting information, or deposited to a public repository. For example, in addition to summary statistics, the data points behind means, medians and variance measures should be available. If there are restrictions on publicly sharing data or code —e.g. participant privacy or use of data from a third party—those must be specified.

Reviewer #1: No: I can't find any mention of it in the main text nor the supplement

We added a reference to this in the text (Data and Code Availability).

Reviewer #2: None

Reviewer #3: Yes

PLOS authors have the option to publish the peer review history of their article (what does this mean?). If published, this will include your full peer review and any attached files.

If you choose “no”, your identity will remain anonymous but your review may still be made public.

Do you want your identity to be public for this peer review? For information about this choice, including consent withdrawal, please see our Privacy Policy.

Reviewer #1: Yes: Michael S Landy
Reviewer #2: No
Reviewer #3: No

Figure Files:

While revising your submission, please upload your figure files to the Preflight Analysis and Conversion Engine (PACE) digital diagnostic tool, https://pacev2.apexcovantage.com. PACE helps ensure that figures meet PLOS requirements. To use PACE, you must first register as a user. Then, login and navigate to the UPLOAD tab, where you will find detailed instructions on how to use the tool. If you encounter any issues or have any questions when using PACE, please email us at figures@plos.org.

Data Requirements:

Please note that, as a condition of publication, PLOS' data policy requires that you make available all data used to draw the conclusions outlined in your manuscript. Data must be deposited in an appropriate repository, included within the body of the manuscript, or uploaded as supporting information. This includes all numerical values that were used to generate graphs, histograms etc.. For an example in PLOS Biology see here: http://www.plosbiology.org/article/info%3Adoi%2F10.1371%2Fjournal.pbio.1001908#s5.

Figure data:
https://figshare.com/articles/dataset/Tracking_the_contribution_of_inductive_bias_to_individualized_internal_models_--_figure_datasets/19620285

Experimental data:

Code for model and figures:
https://github.com/mzperix/asrt-beamsampling

Reproducibility:

To enhance the reproducibility of your results, we recommend that you deposit your laboratory protocols in protocols.io, where a protocol can be assigned its own identifier (DOI) such that it can be cited independently in the future. Additionally, PLOS ONE offers an option to publish peer-reviewed clinical study protocols. Read more information on sharing protocols at https://plos.org/protocols?utm_medium=editorial-email&utm_source=authorletters&utm_campaign=protocols
References:
Review your reference list to ensure that it is complete and correct. If you have cited papers that have been retracted, please include the rationale for doing so in the manuscript text, or remove these references and replace them with relevant current references. Any changes to the reference list should be mentioned in the rebuttal letter that accompanies your revised manuscript.

If you need to cite a retracted article, indicate the article's retracted status in the References list and also include a citation and full reference for the retraction notice.
